# Whole genome sequencing of the fast-swimming Southern bluefin tuna (*Thunnus maccoyii*)

**DOI:** 10.3389/fgene.2022.1020017

**Published:** 2022-11-03

**Authors:** Xiaomeng Zhao, Yu Huang, Chao Bian, Xinxin You, Xinhui Zhang, Jieming Chen, Min Wang, Cancan Hu, Yun Xu, Junmin Xu, Qiong Shi

**Affiliations:** ^1^ College of Life Sciences, University of Chinese Academy of Sciences, Beijing, China; ^2^ Shenzhen Key Lab of Marine Genomics, Guangdong Provincial Key Lab of Molecular Breeding in Marine Economic Animals, BGI Academy of Marine Sciences, BGI Marine, Shenzhen, China; ^3^ Aquatic Breeding Center, BGI Marine, Shenzhen, China; ^4^ BGI Zhenjiang Institute of Hydrobiology, Zhenjiang, China

**Keywords:** southern bluefin tuna (*Thunnus maccoyii*), genome sequencing, assembly, hemoglobin, evolution

## Abstract

The economically important Southern bluefin tuna (*Thunnus maccoyii*) is a world-famous fast-swimming fish, but its genomic information is limited. Here, we performed whole genome sequencing and assembled a draft genome for Southern bluefin tuna, aiming to generate useful genetic data for comparative functional prediction. The final genome assembly is 806.54 Mb, with scaffold and contig N50 values of 3.31 Mb and 67.38 kb, respectively. Genome completeness was evaluated to be 95.8%. The assembled genome contained 23,403 protein-coding genes and 236.1 Mb of repeat sequences (accounting for 29.27% of the entire assembly). Comparative genomics analyses of this fast-swimming tuna revealed that it had more than twice as many *hemoglobin* genes (18) as other relatively slow-moving fishes (such as seahorse, sunfish, and tongue sole). These *hemoglobin* genes are mainly localized in two big clusters (termed as “MNˮ and “LAˮ respectively), which is consistent with other reported fishes. However, Thr39 of beta-hemoglobin in the MN cluster, conserved in other fishes, was mutated as cysteine in tunas including the Southern bluefin tuna. Since hemoglobins are reported to transport oxygen efficiently for aerobic respiration, our genomic data suggest that both high copy numbers of *hemoglobin* genes and an adjusted function of the beta-hemoglobin may support the fast-swimming activity of tunas. In summary, we produced a primary genome assembly and predicted hemoglobin-related roles for the fast-swimming Southern bluefin tuna.

## 1 Introduction

As one migratory fish in the order of Scombriformes, Southern bluefin tuna (*Thunnus maccoyii*; TM) has been an economically important marine species due to its good meat quality. Its length and weight can reach up to 2.45 m and 260 kg (Miller, 1992). The worldwide tuna production continuously increased and reached the highest level to about 7.9 million tons in 2018. Because of overfishing and other human activities, the practical number of the Southern bluefin tuna has decreased significantly, and it has been considered as endangered in the IUCN Red List since 2021 (Collette et al., 2021).

Various tuna species are skilled at continuous and fast swimming (Magnuson, 1973; Blank et al., 2007b). For example, Atlantic bluefin tuna (*T. thynnus*) are reported to reach the highest speed of 54 km per hour (Wardle et al., 1989). Southern bluefin tuna, Atlantic bluefin tuna and Pacific bluefin tuna (*T. orientalis*) are collectively called as bluefin tunas. These bluefin tunas can migrate long distances over vast stretches and tolerate a wide range of temperatures. They can maintain body temperature at 10°C higher than the ambient waters (Blank et al., 2007a; Blank et al., 2007b; Hardison, 2012). They are warm-blooded (rarely among the abundant cold-blooded fishes), enabling them to adapt cold waters and to dive deeper (Bernal et al., 2017). More importantly, bluefin tunas have a high demand for oxygen to keep swimming continuously and at high speeds.

Hemoglobins (Hbs) transport oxygen from the lungs or gills to the rest parts of the body for aerobic respiration in a wide range of animals (Maton et al., 1993). They are usually tetramers with two α and two β subunits (denoted as α_2_β_2_), and each is associated with a heme group. These subunits are similar in size and structure, consisting of seven to eight α-helixes. A total of 17 *hemoglobin* genes (*hb*s) and one pseudogene, consisting of nine α-globins and eight β-globins, were identified in zebrafish (Opazo et al., 2013). *hb* genes in various teleost species are often distributed in two separated clusters, named as MN and LA respectively. MN refers to the left *Methylpurine-DNA Glycosylase (mpg)* and *Nitrogen permease regulator-like 3 (nprl3)* cluster, while LA refers to the right *Leucine carboxyl methyltransferase 1 (lcmt1)* and *Aquaporin-8 (aqp8)* cluster (Hardison, 2008; Opazo et al., 2013).

The genome assemblies of several popular tuna species, such as Pacific bluefin tuna (Nakamura et al., 2013; Suda et al., 2019), Atlantic bluefin tuna (Puncher et al., 2018) and yellowfin tuna (Barth et al., 2017) have been published. However, the detailed genome information of Southern bluefin tuna is still limited except for one assembly uploaded in the public NCBI database (accession no. GCA_910596095.1), which can be used for genomics comparison. In previous studies, the *hb* gene clusters have been well studied in humans and several model fish species, while they remain largely unknown in bluefin tunas. Here, we performed whole genome sequencing of Southern bluefin tuna to provide a valuable genetic resource for identification and characterization of *hb* genes in this fast-swimming tuna species.

## 2 Materials and methods

### 2.1 Sample collection, library construction, and genome sequencing

**FIGURE 1:**
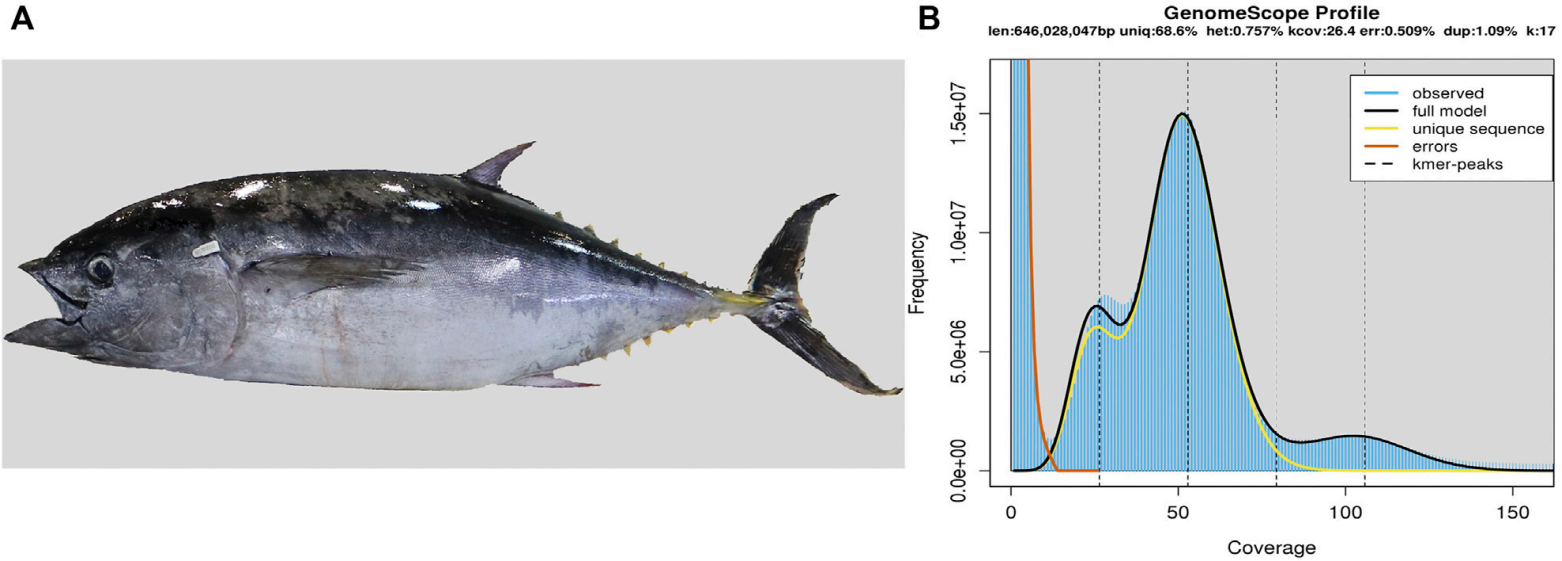
Southern bluefin tuna and its genome size estimation. **(A)** Photo of the sequenced tuna. **(B)** Estimation of the genome size based on the routine k-mer analysis (You et al., 2014). The *X*-axis is the depth of k-mers derived from the sequenced reads, and the *Y*-axis represents the frequency of k-mer depth. Genome size (G) is estimated as follows: G = k_num/peak_depth, where the total number of k-mers (k_num) is 44,309,018,345, and the expected value of k-mer depth (peak_depth) is 52. Therefore, we predicted the genome size of Southern bluefin tuna to be about 852 Mb.

We collected muscle samples and then extracted genomic DNAs from a Southern bluefin tuna (Figure 1A), which was captured from the Great Australian Bight and cultured around the Port Lincoln of Australia. The extracted genomic DNAs were used to construct five libraries following the Illumina protocols, including three short-insert libraries (270 bp, 500 bp, and 800 bp), and two long-insert libraries (2 kb and 5 kb). Subsequently, based on the routine shotgun sequencing strategy (You et al., 2014), whole genome was sequenced on Hiseq 2500 and Hiseq X-ten platforms (Illumina, San Diego, CA, United States). Raw reads were filtered by SOAP filter (v2.2) (Luo et al., 2015) before assembly.

### 2.2 Genome size estimation and *de novo* genome assembly

We conducted a 17-mer distribution analysis to estimate the target genome size based on the short-insert libraries (Liu et al., 2013; Vurture et al., 2017). We calculated the genome size (G) using the following formula as reported before (You et al., 2014): G = k_num/peak_depth (see more explanations in the legend of Figure 1B).

We employed Platanus v1.2.4 (Tokyo Institute of Technology, Tokyo, Japan) with an optimized parameter “-k 29” to obtain a *De Bruijin* assembly by using Illumina paired-end reads (Kajitani et al., 2014). We generated a total of 1,564,055 contigs (Supplementary Table S5), and then we constructed scaffolds and filled the gaps of intra-scaffolds using the reads of short-insert sizes (270 bp, 500 bp, and 800 bp) with Platanus v1.2.4. To further estimate the completeness of our assembly, the BUSCO (Benchmarking Universal Single-Copy Orthologs; version 1.22) software was applied based on the actinopterygii_odb9 database.

### 2.3 Repeat sequence analysis

To analyze the assembled genome for repeat sequences, we first employed Tandem Repeats Finder (version 4.07) to search tandem repeats (Benson, 1999). Secondly, we employed RepeatMasker (version 4.0.6) and RepeatProteinMask (version 4.0.6, an updated software in the RepeatMasker package) to detect known transposable elements (TEs) based on the public Repbase TE library (release 21.01) (Jurka et al., 2005; Tarailo-Graovac and Chen, 2009; Bao et al., 2015). Thirdly, we applied RepeatModeler (version 1.0.8) and LTR_FINDER (version 1.0.6) with default parameters (Xu and Wang, 2007; Abrusan et al., 2009) to generate a *de novo* repeat library. Finally, we employed RepeatMasker to identify and classify homologous repeats against this *de novo* repeat library.

### 2.4 Genome annotation and evaluation

We utilized three different approaches to annotate detailed structures of the predicted genes in our assembled genome, including *de novo* prediction, homology-based prediction, and transcriptome-based annotation (You et al., 2014). For the *de novo* prediction, we employed AUGUSTUS (version 3.2.1) and GENSCAN (version 1.0) to identify protein-coding genes with the repeat-masked genome (Burge and Karlin, 1997; Stanke et al., 2006). For the homology-based prediction, we aligned the homologous proteins of seven representative fish species, including zebrafish (*Danio rerio*), threespine stickleback (*Gasterosteus aculeatus*), Nile tilapia (*Oreochromis niloticus*), medaka (*Oryzias latipes*), Japanese pufferfish (*Takifugu rubripes*), green spotted pufferfish (*Tetraodon nigroviridis*), and Atlantic cod (*Gadus morhua*) (Ensembl 83 release), to the repeat-masked genome using tblastn (Blastall version 2.2.26) with an e-value ≤ 1e-5 (Mount, 2007; Cunningham et al., 2015). Subsequently, Solar (version 0.9.6) and GeneWise (version 2.4.1) were executed to define the potential gene structures for all alignments (Birney et al., 2004; Li et al., 2010). Next, we employed TopHat (version 2.0.13) to map RNA reads and ensemble genomes, and then we applied Cufflinks (version 2.2.1) to assemble and merge transcripts using the accepted hits of TopHat (Trapnell et al., 2009; Trapnell et al., 2010). Finally, we combined the above-mentioned three datasets to obtain a consistent and comprehensive gene set by GLEAN (version 1.0) (Elsik et al., 2007).

The predicted coding proteins of the Southern bluefin tuna were aligned against KEGG (Kyoto Encyclopedia of Genes and Genomes), SwissProt and TrEMBL databases for prediction of functions and pathways by using BLASTP (Kanehisa and Goto, 2000; Boeckmann et al., 2003). Subsequently, we applied InterProScan (version 5.16-55.0) to identify functional motifs and domains through the Pfam, PRINTS, ProDom and SMART databases (Attwood, 2002; Letunic et al., 2004; Bru et al., 2005; Hunter et al., 2009; Finn et al., 2014).

### 2.5 Genomics comparison of different specimens of southern bluefin tuna

There is a chromosome-level genome assembly of Southern bluefin tuna in NCBI (GCA_910596095.1) without publication of related paper; in order to compare our genome assembly with this public version, we reordered our scaffolds into pseudochromosomes by RaGOO (Alonge et al., 2019) using the public version as the reference. Then, genome-wide alignments were performed using minimap (Li, 2016), and the best homology segments were selected for visualization by SyRI (Goel et al., 2019). The repeat content and gene annotation of the public assembly were also retrieved from NCBI. Numbers of genes and exons of each gene were counted, and length distribution of the CDS, exons and introns were plotted by the routine R package.

### 2.6 Identification of *hemoglobin* genes

We used zebrafish hemoglobin protein sequences, downloaded from the Ensemble database (Supplementary Table S1), as the queries to extract *hb* genes in the genome assemblies of Southern bluefin tuna (this study), Pacific bluefin tuna, swordfish, sailfish, Atlantic cod, tiger tail seahorse, greater amberjack, yellowtail amberjack, tongue sole, large yellow croaker, European seabass, zebrafish, and ocean sunfish (Supplementary Table S2). We constructed a local database for each fish, and performed tblastn (v2.2.28) to align these sequences with an e-value cutoff of 10^–5^ for localizing the orthologs of these queries (Mount, 2007). Subsequently, we employed Exonerate (v2.2.0) to evaluate the alignment results within the entire encoding regions (Slater and Birney, 2005).

To further characterize hemoglobin structures, we downloaded sea lamprey, spotted gar and human hemoglobin amino acid sequences (Supplementary Table S1) from the public Ensemble database. In addition, we used sequences of *hb* genes and 12 neighboring genes from the zebrafish genome to determine syntenic correlations among Southern bluefin tuna and other examined fishes. To evaluate the conservation of *hb* genes, we used five genes upstream plus two genes downstream of the MN cluster, and three genes upstream plus two genes downstream of the LA cluster for a synteny analysis (Opazo et al., 2013), which included *mgrn1b (Mahogunin ring finger 1b)*, *aanat2 (Arylalkylamine N-acetyltransferase 2)*, *rhbdf1a (Rhomboid family member 1a)*, *mpg*, *nprl3*, *kank2 (KN motif and ankyrin repeat domain-containing protein 2)*, *dock6 (Dedicator of cytokinesis 6)*, *mgrn1a*, *foxj1b (Forkhead box j1b)*, *rhbdf1b*, *aqp8*, *lcmt1*, and *arhgap17a (Rho GTPase-activating protein 17a)* (see detailed accession numbers in Supplementary Table S1). They are conserved in teleosts, thus we used their sequences to search for corresponding syntenic locations. We obtained the related genome data from NCBI as mentioned above. Subsequently, the routine strategy of protein sequences aligned to nucleotides was employed to examine these extracted synteny genes in various fish genomes by using tblastn (Blastall version 2.2.26) with an e-value ≤ 1e-5 (Mount, 2007). Next, Solar (version 0.9.6) was executed to define the potential gene structures for all alignments (Li et al., 2010). Finally, we draw the figures with GSDS (Gene structure display server v2.0; Hu et al., 2014).

### 2.7 Phylogeny of *hemoglobin* genes

These *hb* genes in Southern bluefin tuna were then deduced as protein sequences for phylogenetic analyses. Then, we predicted their best nucleotide substitution model using IQ-TREE (version 1.6.10) under the Bayes information criterion (BIC) (Nguyen et al., 2015; Kalyaanamoorthy et al., 2017). Parameters within the best nucleotide substitution model of WAG + G4 (bluefin tunas) was applied into PhyML (version 3.0) to construct phylogenetic trees with the method of maximum likelihood (ML) and 4,000 replicates for evaluation of their branch supports (Guindon et al., 2010). We selected the *hb* genes in Southern bluefin tuna, Pacific bluefin tuna, swordfish, greater amberjack, and spotted gar, for the phylogenetic analysis using jModeltest2 under AIC with the best nucleotide substitution model of GTR + I + G.

### 2.8 Sequence alignment of fish hemoglobin proteins

Multiple sequence alignment of these predicted *hb* genes was performed with the Muscle module in MEGA (version 7.0) (Kumar et al., 2016). Southern bluefin tuna, Pacific bluefin tuna, swordfish, greater amberjack were chosen to align with zebrafish and human hemoglobin proteins. The final alignment results were colorize by TEXshade (Beitz, 2000).

## 3 Results

### 3.1 Summary of the genome assembly and annotation

We obtained 173.19 Gb of raw reads and 135.80 Gb of clean reads for genome assembly of the Southern bluefin tuna (Supplementary Table S3). The estimated genome size of Southern bluefin tuna was 852 Mb by the k-mer analysis (Figure 1B; Supplementary Table S4), and the final assembly was 806.54 Mb in length, with contig and scaffold N50 values of 67.38 kb and 3.31 Mb, respectively (Table 1; Supplementary Table S5). The BUSCO analysis shows that the assembled genome contains 4,390 (95.8%) complete and 105 (2.3%) duplicated BUSCOs, suggesting that the current assembly was qualified for downstream analyses.

**TABLE 1:** Summary of the genome assembly and annotation for Southern bluefin tuna.

Genome assembly	Data
Contig N50 size (kb)	67.38
Scaffold N50 size (Mb)	3.31
Assembled genome size (Mb)	806.54
Genome coverage (×)	168.37
The longest scaffold (bp)	14,783,706
Genome annotation	Data
Protein-coding gene number	23,403
Annotated functional gene number	22,485 (96.08%)
Unannotated functional gene number	918 (3.92%)
Repeat sequences	29.27%

We then annotated repeat elements and protein-coding genes in the assembled genome. Repeat sequences accounted for 29.27% (236.1 Mb) of the assembled genome, including 9.87% DNA, 5.04% LINE, 0.28% SINE, 1.65% LTR, (Supplementary Tables S6, S7). A total of 23,403 protein-coding genes were annotated, with an average of 9.82 exons and 1800-bp coding sequences per gene (Supplementary Table S8). Based on the public KEGG, SwissProt and TrEMBL databases, 96.08% (22,485) of the predicted genes were assigned to at least one function (Supplementary Table S9). To estimate the quality of our annotated genes, we determined a total of 91.7% complete BUSCOs.

### 3.2 High-throughput identification of *hemoglobin* genes

**FIGURE 2:**
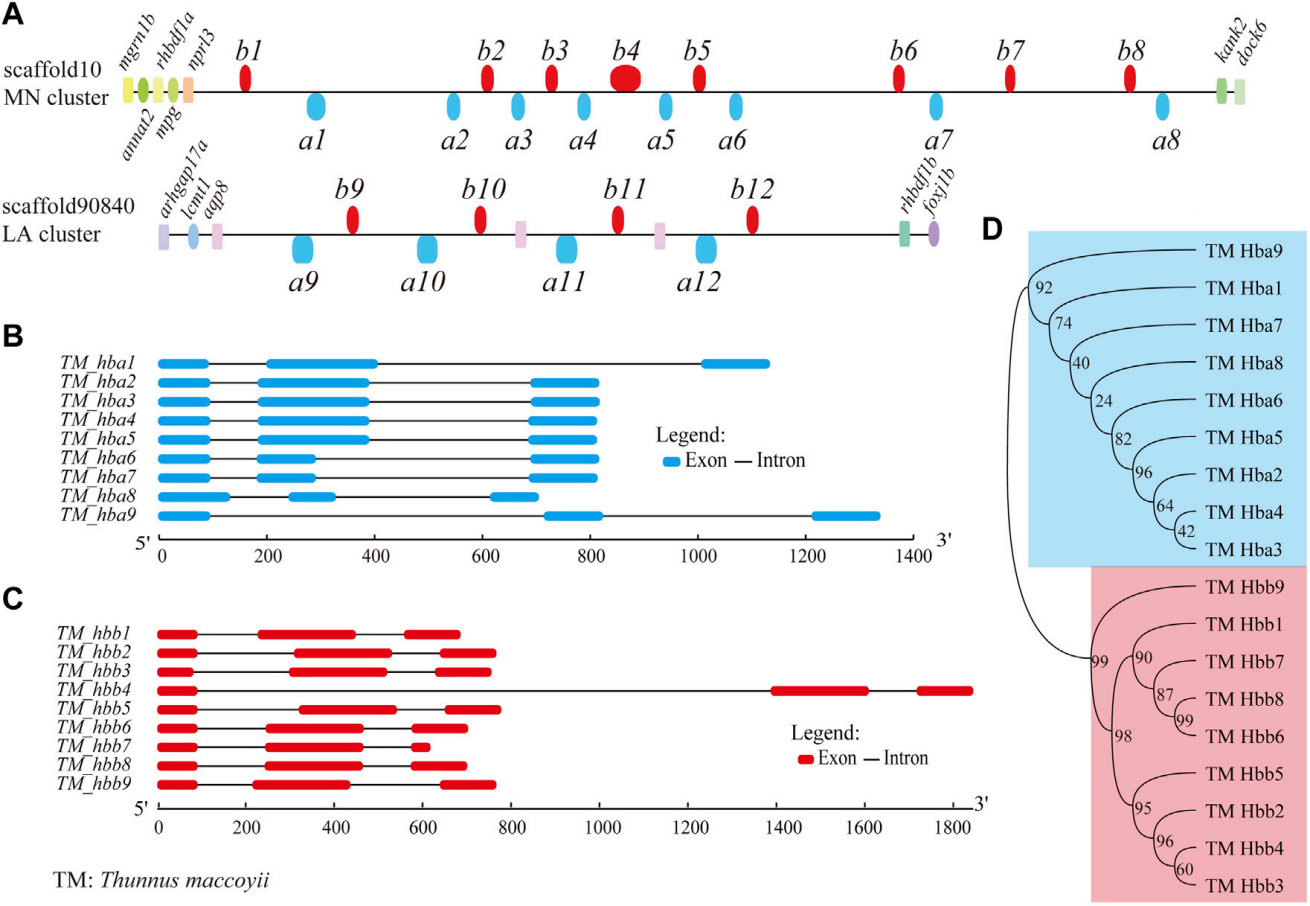
*Hemoglobin* genes in the Southern bluefin tuna genome. **(A)** Genome-wide distribution of *hb* genes in Southern bluefin tuna (*T. maccoyii*, TM). *hb* genes above the line are encoded by forward strands, while those below the line were encoded by reverse strands. We added the *aqp8-hba-hbb* repeats with six more *hb*s (between *a10* and *b12*) in the LA cluster, since they are present in the public NCBI version. **(B,C)** Detailed structures of *hb* genes (classified into *hba* and *hbb*; marked in blue and red, respectively). **(D)** Phylogenetic evolution of *hb* genes.

We selected *hb* genes with the main aim to study their potential roles for continuous swimming with a high speed. A total of 18 *hb* genes distributing on two scaffolds were identified from the assembled genome, including nine α*-hemoglobin* genes (*hba*) and nine *β-hemoglobin* genes (*hbb*) (Figure 2A; Table 2). All these *hb* genes were composed of three exons, which are classic for *hb* genes (Figures 2B,C). Their total length ranged from 687 bp to 1,846 bp (143–147 amino acids), although the lengths of introns were variable.

**TABLE 2:** Copy numbers of *hemoglobin* (*hb*) genes in the examined genomes.

Species		*hba*	*hbb*	MN cluster	LA cluster	Total	Note*
Scientific name	Common name						
*Thunnus maccoyii*	Southern bluefin tuna	9	9	16	2	18	Fast
*Thunnus orientalis*	Pacific bluefin tuna	9	7	12	4	16	Fast
*Xiphias gladius*	Swordfish	8	8	14	2	16	Fast
*Danio rerio*	Zebrafish	8	8	13	3	16	Ref.
*Seriola dumerili*	Greater amberjack	6	7	11	2	13	Fast
*Larimichthys crocea*	Large yellow croaker	8	5	11	2	13	Fast
*Seriola aureovittata*	Yellowtail amberjack	5	7	10	2	12	Fast
*Dicentrarchus labrax*	European seabass	6	5	8	3	11	Fast
*Gadus morhua*	Atlantic cod	4	5	4	5	9	Slow
*Mola mola*	Ocean sunfish	5	3	4	4	8	Slow
*Cynoglossus semilaevis*	Tongue sole	4	3	2	5	7	Slow
*Hippocampus comes*	Tiger tail seahorse	3	1	2	2	4	Slow
*Homo sapiens*	Human	5	5	5	5	10	Ref.
*Lepisosteus oculatus*	Spotted gar	3	4	—	—	7	Ref.
*Petromyzon marinus*	Sea lamprey	—	—	8	2	10	Ref.

^*^Fast: fast-swimming fishes; Slow: slow-swimming fishes; Ref.: reference species.

Almost all *hb* genes (except for two) were localized in the MN cluster (scaffold10), displaying in both forward and reverse orientations (upper Figure 2A). They were named from left (close to *nprl3*) to right sequentially as follows: *hbb1*, *a1*, *a2*, *b2*, *a3*, *b3*, *a4*, *b4*, *a5*, *b5*, *a6*, *b6*, *a7*, *b7*, *b8*, and *a8*. The remaining two *hemoglobin* genes, named *hba9* and *hbb9,* were identified in the LA cluster (scaffold90840) (lower Figure 2A). Neighboring genes to both MN and LA clusters were also annotated (Figure 2A).

The phylogenetic tree shows that the two types of Southern bluefin tuna *hb* genes formed two independent groups as expected, i.e., the clades *hba* and *hbb* (Figure 2D). Interestingly, *hba2, a3, a4*, and *a5* had similar gene structures with similar lengths of exons and introns (Figure 2B), and they were close in evolution (top panel in Figure 2D). Similar phenomenon was also found for *hba1, a6, a7,* and *a8* (Figure 2D). Such a topology supports a common evolutionary origin of the two types of *hb* genes, and each type underwent expansion through a series of gene duplications and independent evolution (Storz, 2016; Lei et al., 2021).

### 3.3 Genomic comparisons of *hemoglobin* genes in representative teleost species

**FIGURE 3:**
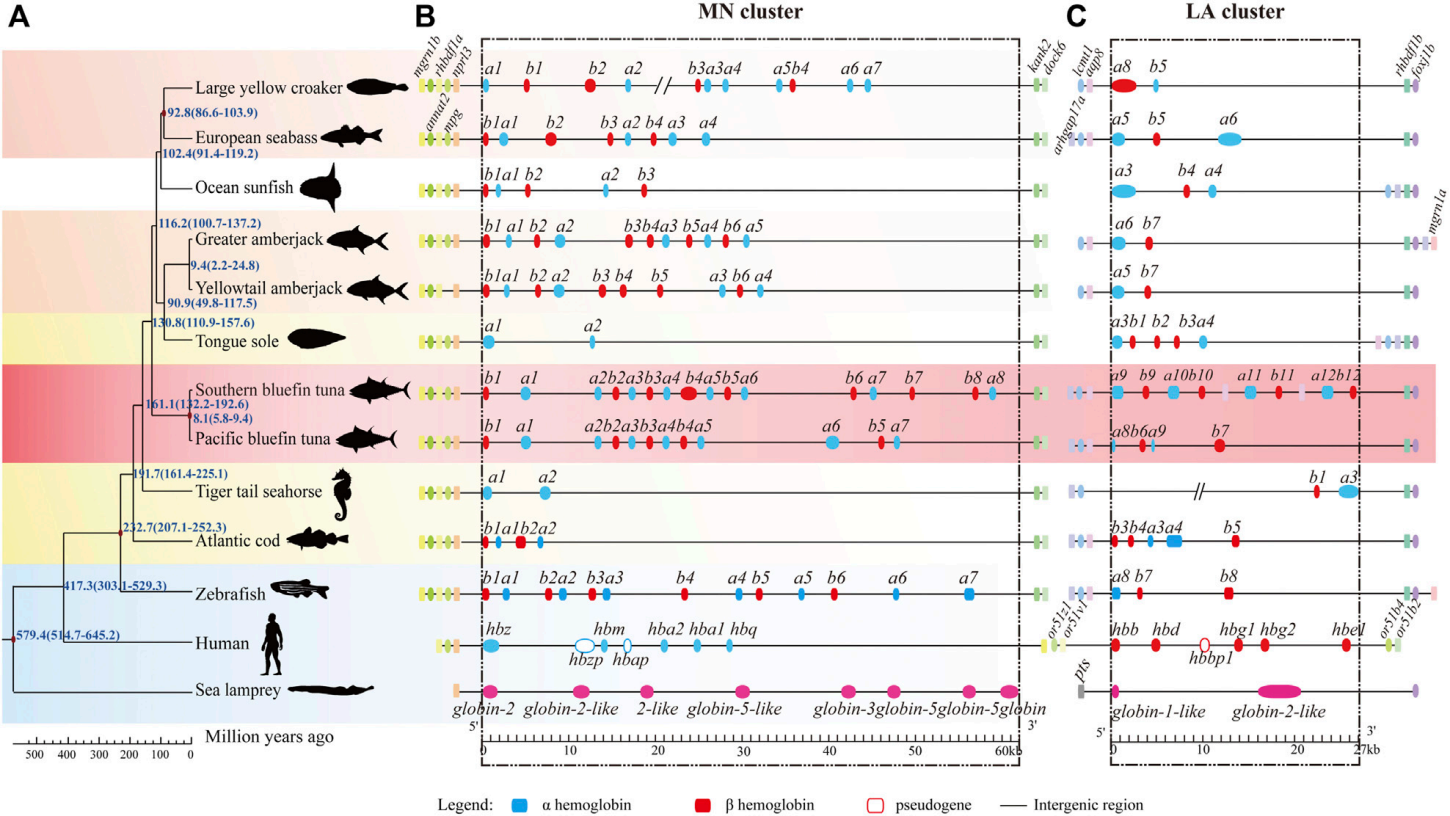
Collinearity of *hemoglobin* genes in representative fish genomes. **(A)** Phylogenetic tree of representative species, ranging from the jawless vertebrate lamprey to mammalian human with a time scale for divergences (millions of years ago, MYA). Sea lamprey (*Petromyzon marinus*) was set as the outgroup. Background was colored as red for two bluefin tunas, light red for other fast-swimming fishes, yellow for slow-swimming fishes, and blue for the reference species. **(B,C)** *hb* genes and their neighboring genes. Blue and red boxes represent *hba* and *hbb* genes, respectively. Neighboring genes were named at their first appearance on the top of the diagram. We added the *aqp8-hba-hbb* repeats with six more *hb*s (between *a10* and *b12*) in the LA cluster of Southern bluefin tuna, since they are present in the public NCBI version.

To investigate *hb* genes among various vertebrates, a collinearity analysis of them as well as their neighboring genes was performed using the genomes of sea lamprey, human, spotted gar and 11 teleosts (Table 2). We chose these representatives because 1) their genome assemblies are of high quality; 2) some are model species; 3) they are representatives through the fish tree of life; 4) they are fast-swimming as tunas or slow-swimming in contrast. The conserved genes located in upstream and downstream regions of *hb*s were used as markers to distinguish MN and LA clusters (Figure 3). Our results showed that the genomic positions of teleost *hb* gene clusters were highly conserved but the gene number and order in the clusters varied between species, especially in the MN cluster. All fish MN clusters contained the five genes (*marn1b, annat2, rhbdf1a, mpg*, and *nprl3*) upstream and two genes (*kank2* and *dock6*) downstream to the hemoglobin duplicates (Figure 3B). However, LA clusters were relatively diverse among various fishes. Most fishes possessed two genes (*aqp8* and *lcmt1*) upstream and two genes (*rhbdf1b* and *foxj1b*) downstream to the hemoglobin family, with exceptions for ocean sunfish, tongue sole and tiger tail seahorse (Figure 3C). We cannot detect a complete *hba9* sequence in the LA cluster of zebrafish, therefore only 15 *hb* genes of zebrafish were presented in Figure 3.

After identification of *hb* genes in all examined fishes, we found that the fast-swimming tunas and billfishes contained more *hb* genes than other fishes, suggesting adaptation by obvious expansion of the *hb* gene family in these lineages (17–18 copies; see Table 2; Figure 3). In contrast, those languid species (such as seahorses, tongue sole and sunfish) had much fewer *hemoglobin* gene copies (only 4–8; see more details in Table 2; Figure 3). These interesting findings suggest that the copy number variation of *hb* genes may be involved in the fish swimming activity; it seems that the more *hb* genes a fish contains, the faster it can swim.

### 3.4 Phylogenetic relationships among representative teleost fishes

**FIGURE 4:**
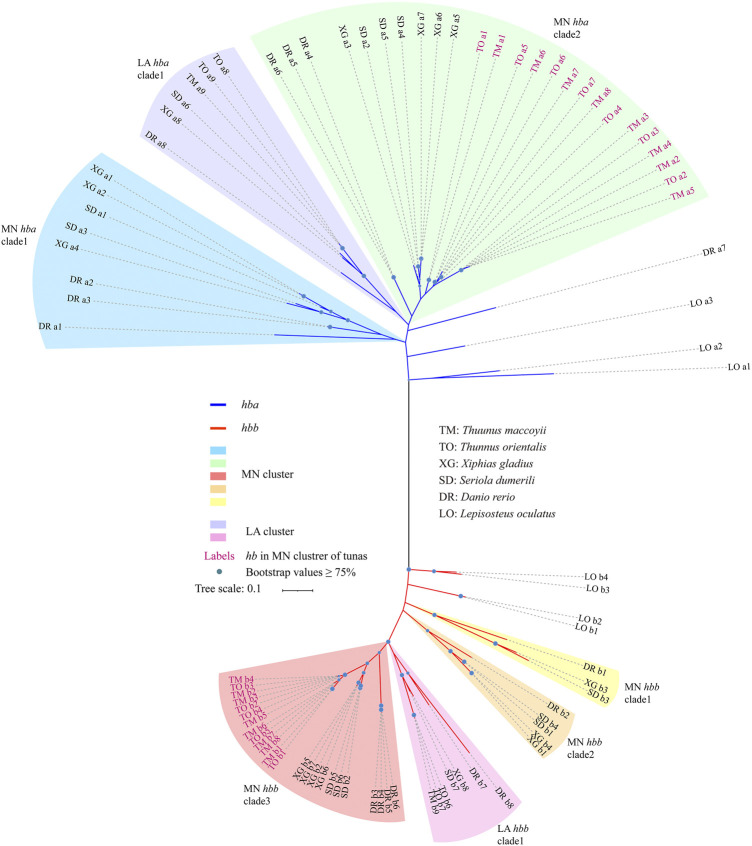
Phylogenetic evolution of *hemoglobin* genes in six representative species. Branch colors represent different clades of α*-hemoglobin* genes (in blue) and β*-hemoglobin* genes (in red). Note that *hemoglobin* genes in the MN and LA clusters were marked by different colors.

To investigate the evolutionary history of fish *hb* genes, we constructed an unrooted phylogenetic tree using the coding sequences from five teleosts and spotted gar (Figure 4). These *hb* genes were grouped into two clades of α (with blue branches) and β (with red branches) with seven *hb* genes of spotted gar as the ancestors (Feng et al., 2014), and clustered into discrete MN and LA clades (Figure 4). The hemoglobin protein in sea lamprey is a monomeric molecule (Hendrickson and Warner, 1971; Schwarze et al., 2014), but in jawed vertebrates it is a tetrameric molecule with two α and two β subunits. It is believed that the proto *hb* gene was duplicated to form α and β combined hemoglobins (Goodman et al., 1975; Hoffmann et al., 2012; Storz et al., 2013). The phylogenetic tree of *hbb* genes were grouped into four well-supported subclades, and those genes associated with the LA and MN clusters were resolved as paraphyletic (Figure 4). The genealogical relationships among the orthologous sequences were consistent with known organismal relationships.

All *hbb* genes derived from LA clusters were clustered in one clade (Figure 4). The LA *hbb* subclades possessed orthologous sequences derived from each species. The relationship in the MN cluster was more complicated, since the cluster was divided into three subclades. The *hbb* genes of tunas [*T. maccoyii* (TM) and *T. orientalis* (TO)] formed an independent clade (MN clades 3), while in the other teleost species they were in each of the three MN clades. In this study, the MN cluster of both TM and TO possessed a more extensive and varied composition of *hb* genes when compared with other examined fishes. Similar to the *hbb* genes, *hba* genes were arranged into three subclades, with genes from the MN cluster forming a paraphyletic group relative to those from the LA cluster. The *hba* genes derived from TM and TO in the MN cluster formed the sister group with the other species in MN clade 2 (Figure 4).

### 3.5 Sequence alignment of fish hemoglobin proteins

**FIGURE 5:**
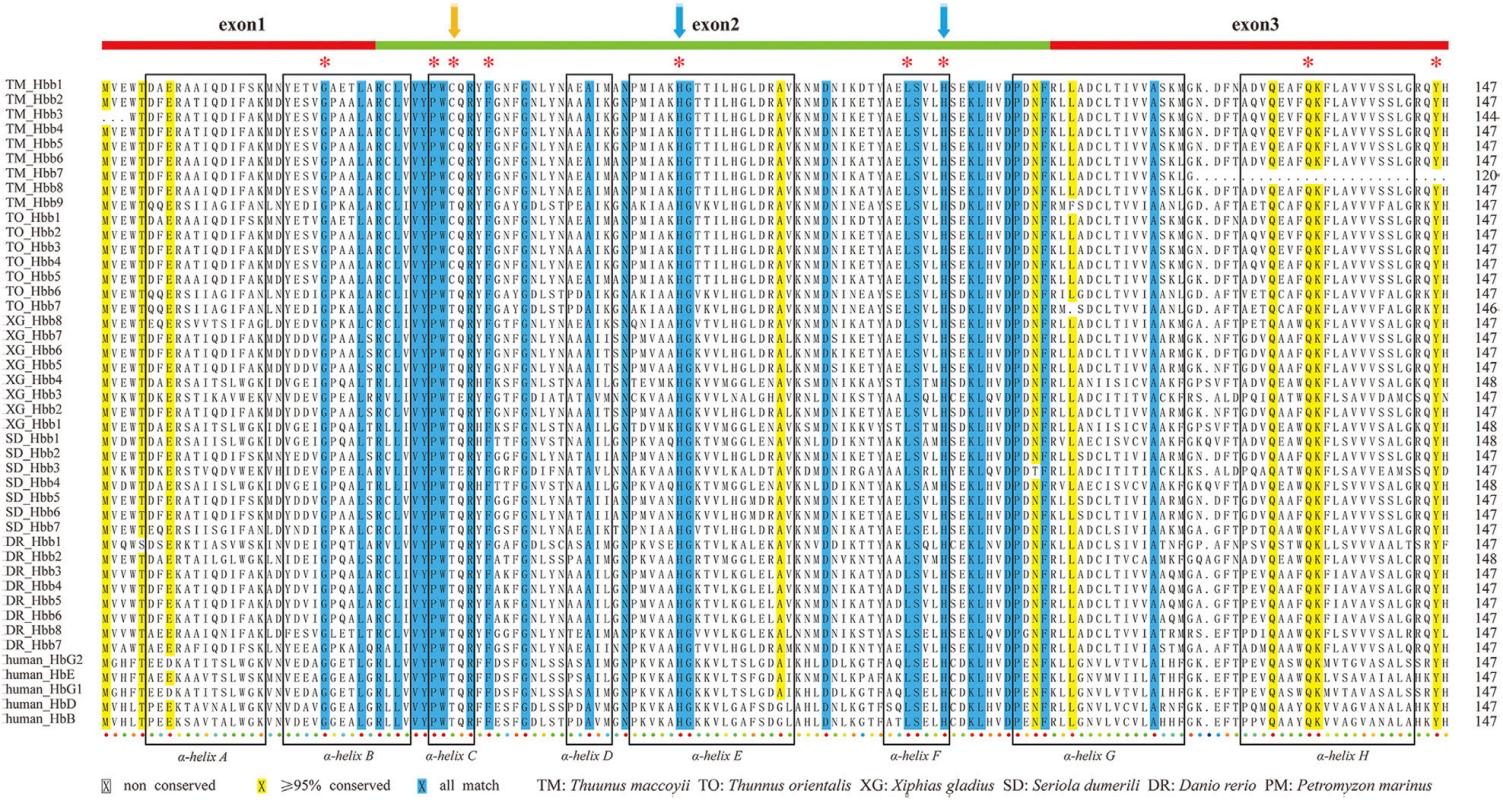
ClustalX alignments of β-hemoglobins from six representative species. Eight boxes with α-helical segments were named as A ∼ H. Conserved distal and proximal histidine residues were marked by asterisks. Two blue arrows indicate the distal and proximal histidines for putative involvement in the heme binding. The orange arrows marked the loci that are unique to tunas (cysteine), which are different from those in other species (threonine). Alignments of α-hemoglobins are also provided in Supplementary Figure S4 for comparison.

Complete amino acid sequences of *hb* genes from representative teleosts and human were aligned for comparing the gene structures and conservation. Protein sequence alignments showed that both α (Supplementary Figure S4) and β (Figure 5) hemoglobins were highly conserved, although the two types varied slightly in several ways. First, the β-hemoglobins contained eight α-helixes (named as A ∼ H), with a length of 147 or 148 amino acid (aa) long, while the α-hemoglobins were 141–144 aa in length, losing the α-helix D. Second, histidines in helixes E and F, the putative binding sites to heme (Bolton and Perutz, 1970; Blouquit et al., 1984; Frischknecht et al., 1996), were completely consensus in all examined β-hemoglobins (blue arrows in Figure 5), whereas in one zebrafish α-hemoglobin (*hba8*), the histidine in helix E was mutated to glutamine. Last but most importantly, we observed an interesting substitution of threonine (Thr, T) to cysteine (Cys, C) at the position 39 of β-hemoglobin in the MN cluster, and such a point mutation (Figure 5; Supplementary Figure S5) was found in all reported tuna genomes (*T. maccoyii, T. orientalis, T. albacares*; Supplementary Table S10). Previous reports have proved that this conserved site was strongly related to the oxygen affinity (Blouquit et al., 1984; Frischknecht et al., 1996), which inspires us to speculate that this linages-specific mutation was potentially conducive to oxygen transport for their high speed and continue swimming in tunas.

## 4 Discussion

### 4.1 Comparison of reported tuna genome assemblies

There are eight species in the genus *Thunnus* (including *T. alalunga*, *T. albacares*, *T. atlanticus*, *T. maccoyii, T. obesus*, *T. orientalis*, *T. thynnus*, and *T. tonggol*), and four of them had been sequenced and assembled (Supplementary Table S10). This study assembled an 806-Mb draft genome for Southern bluefin tuna, which is an indispensable supplement to the comparative genomic study of this important genus.

Here, we curated all available tuna genome data and made some comparisons. The sizes of these tuna genomes were mainly between 720 Mb and 830 Mb (Supplementary Table S10), with a similar BUSCO completeness, with an exception for *T. thynnus* (Puncher et al., 2018). In fact, the reported genome size of *T. thynnus* reached 944 Mb, but the downloaded genome from NCBI was only 648 Mb with 36.9% complete BUSCOs, which may influence the quality of this genome assembly for comparative analysis. That is likely why some *hb* genes and their flanking genes were not detectable in one cluster of *T. thynnus*. The GC content of the four tuna genomes were similar (Supplementary Table S10), supporting relative reliability of our genome assembly.

Based on the public genome data of a different specimen of Southern bluefin tuna from the NCBI, we anchored our scaffold-level assembly into 24 pseudochromosomes too. A total of 791.3 Mb was anchored, corresponding to 98.1% of our assembled genome with a high coverage (93.92%) of the public assembly (Supplementary Table S12). There were 716.59-Mb syntenic regions between the two genome versions. Subsequently, we performed a collinearity analysis of these two assemblies, which shows perfect one-to-one synteny relationship between each pair of chromosomes, with exceptions of a few translocations, inversions, and duplications (Supplementary Figure S1). After compared the two genome assemblies of Southern bluefin tuna, we found that the distributions of exon length, intron length, exon number and CDS length were similar (Supplementary Figure S2). We also compared the repeat contents and gene sets of the two assemblies. It seems that our genome assembly has a slightly higher repeat content (29.27%) than the public version (26.07%), but both assemblies have an approximate number of predicted genes (23,403 vs. 24,659), and similar distributions of exon number, exon length, intron length and CDS length (Supplementary Figure S2). All these results suggest that our present assembly is somehow comparable to the public chromosome-level version.

### 4.2 Copy number variation of fish *hemoglobin* genes

The *hb* gene repertoire of Southern bluefin tuna consisted of nine *hba* and nine *hbb* genes. With these 18 genes in total, the Southern bluefin tuna had the largest number of *hb* genes among all examined diploid fishes so far (Table 2). According to previous report, tunas and billfishes can reach speeds of 50 km per hour (Wardle et al., 1989; Wu et al., 2021). Tongue sole is a benthic fish and rarely swims speedily (Chen et al., 2014). Sunfish can swim at continuous speeds of 1.4 km/h–2.5 km/h (Pope et al., 2010). The swim speed of Atlantic cod is at 0.72 km/h–1.44 km/h (Blaikie and Kerr, 1996). Without demands of speed, the more languid seahorses stay still for most of the time (Blake, 1976). Billfishes, maintaining continuous swimming activities and high speed, and similar in functional morphology to tunas (Wu et al., 2021), have a relatively high copy number of *hb* genes as well. Our comparative genomics analysis indicated that this fast-swimming tuna and other active fishes (such as pacific bluefin tuna, swordfish, and greater amberjack) had more *hb* genes than those relatively languid fishes (such as seahorse, sunfish, and tongue sole; see more details in Table 2; Figure 3).

Unlike most mammals, fishes have multiple *hb* genes and their copy number variation has been discussed in several studies. These previous reports suggest that variations in *hb* copy number may be related to physiological differences in blood oxygen transport and aerobic energy metabolism (Hoffmann and Storz, 2007; Rutjes et al., 2007). As previously discussed, the diversity of hemoglobin in fishes is related to their adaptation to different environmental conditions or habitats (Verde et al., 2006). It has been proposed that less *hb* gene copies in polar fishes were related to their sluggish style of life and lower metabolism (Verde et al., 2006; Verde et al., 2007; Dettaï et al., 2008). In a previous report of channel catfish, the embryonic *hb* genes showed importance in coping with the low oxygen conditions under heat stress (Feng et al., 2014). In contrast, fishes with higher metabolism and diverse environmental conditions, such as Atlantic salmon, tend to have more *hb* copies (Quinn et al., 2010). Tunas always have high rates of energy turnover and metabolism to maintain fast and continuous swimming in an open pelagic environment (Brill, 1996; Korsmeyer et al., 1996; Jensen, 2001), thus more oxygen is absolutely necessary for the high-efficiency metabolism. Improved repertoire of more *hb* genes in these fast-swimming fishes may be beneficial for oxygen transportation and energy metabolism (Hoffmann et al., 2010; Opazo et al., 2013).

### 4.3 Expansion of *hemoglobin* genes in the tuna lineage

To figure out how the *hb* gene family is expanded in Southern bluefin tuna, we performed subsequent synteny and phylogenetic analyses. Our data show that almost all tuna *hb* genes (16 out of 18) were localized in the same MN cluster, indicating that the *hb* expansion in tuna is a result of emerging *hb* genes in the MN instead of the LA cluster. Specially, those genes in the MN cluster encoding α subunits were clustered together with the *β-hemoglobin* genes, appearing as pairs of one α- plus one β-globin genes, such as *hba2-hbb2* and *hba3-hbb3* (Figure 2A). Such an alternate arrangement of *hb* genes was reported in some other fish species, which is benefit for efficient oxygen transcription (Flint et al., 2001; Maruyama et al., 2004; Hardison, 2008; Negrisolo et al., 2008; Quinn et al., 2010; Opazo et al., 2013; Feng et al., 2014; Storz et al., 2020; Lei et al., 2021).

The expansion of *hb* genes in the MN cluster was not only valid in the Southern bluefin tuna, but also in other tunas such as the yellowfin tuna and Pacific bluefin tuna (Supplementary Figure S3). However, the LA clusters among the tunas were relatively variable. In addition to the four *hb* genes, the *T. maccoyii* and *T. albacares* assemblies had extra copies of *aqp8-hba-hbb* repeats downstream, which has not been identified in any other fishes before. The public version assembly probably recovered a full LA cluster, we therefore added the *aqp8-hba-hbb* repeats in Figures 2, 3 with the public version as the reference.

Tandem duplicates often have similar coding sequences owing to interparalog gene conversion. A balanced and synchronous expression of α and β subunits is reported to be good for efficient production of hemoglobin tetramers (Chan et al., 1997; Miyata and Aoki, 1997; Chu et al., 2006; Weatherall and Clegg, 2008) with the same transcriptional polarity, thereby increasing the capacity to transport oxygen. Expansion of the α-β *hemoglobin* gene pairs is supposed to adapt for high speeds and continues swimming for various environments in tunas. Such expansion of tuna *hb* genes in the MN cluster was further supported by the predicted phylogeny (Figure 4), which also provides insights into the origin of those newly emerged *hb* genes.

### 4.4 Mutation of β39Thr-Cys in tunas

We observed an interesting substitution of Thr to Cys at the position 39 of β-hemoglobins in MN clusters of all reported tuna genomes (Figure 5; Supplementary Figure S5). As reported before, the highly conserved residue Thr-β39 is often contacted to the heme at the vinyl side chain of the heme ring, thus it is essential for hemoglobin function (Bolton and Perutz, 1970; Frischknecht et al., 1996). Other studies proved that Thr-α38 is also invariant in mammals (Dickerson and Geis, 1983), and it is important for the oxygen binding property (Hashimoto et al., 1993).

In human, there are three known types of mutations at the position 39 in β-hemoglobins, including Hb Hazebrouk (Thr–Pro) and Hb Grove City (Thr–Ser) that may decrease oxygen affinity and cause hemolytic anemia (Blouquit et al., 1984; Taliercio et al., 2013), and Hb Hinwil (Thr–Asn) that in contrast can elevate oxygen affinity but cause a remarkable reduction of cooperativity (Frischknecht et al., 1996). We analyzed the 12 species in Table 2 (see Supplementary Figures S5, S6) and selected more eleven fishes for further analysis, which represented nine orders and near the tunas in the tree of life (Supplemntary Table S11). We confirmed that the mutation happened only in the MN cluster of all tunas and one possibly in the LA cluster of *Boleophthalmus pectinirostris* [BP, one of three selected mudskippers; (You et al., 2014)]. Whether it is true in BP and other mudskippers are worthy of more investigations.

Analysis of the coding genes of hemoglobins in various fishes showed that this substitution was ACT/ACC/ACG/ACA to TGC/TGT (Supplementary Figure S6). In the MN cluster of tunas, the codons for Cys are TGC (27/29) and TGT (2/29). While in other fishes and spotted gar, the codons for Thr are ACT (48/67), ACC (14/67), ACG (3/67) and ACA (2/67) (Supplementary Table S11). In the LA cluster of fishes, the codons for Thr are ACT (37/41), ACC (3/41), and TGT (1/41, only in mudskippers). When this site mutates through the path of Thr- > Ser- > Cys, the first position takes A- > T, the second goes by C- > G, and the third position retains C or T. Because we could not detect the codons for Ser (TCT/TCC/TCG/TCA) in all examined fishes at this site, we are not sure that disadvantageous mutations might have occurred. In some previous reports, cysteine residues of human hemoglobins mainly function for local nitric oxide regulation as needed (Pawloski et al., 2001; Feng et al., 2014). Since beta39Thr- > Cys substitution is not observed in other selected fast-swimming fishes (eg, swordfish), it may not be critical for the fast-swimming ability, while it is still a unique position in tunas. This cysteine substitution at the position 39 in tunas may therefore adjust functions of the hemoglobins, which possibly benefits for aerobic respiration and supports the fast-swimming activities of tunas.

## 5 Conclusion

We performed whole genome sequencing and assembled a draft genome for Southern bluefin tuna, based on which a total of 18 *hemoglobin* genes were identified. In detail, there were eight *hba* and eight *hbb* genes in the MN cluster and one *hba* and one *hbb* in the LA cluster. Genomic comparison analysis shows that this fast-swimming tuna had the largest *hemoglobin* gene number among all examined diploid fishes. The phylogenetic tree supports a common origin of the two types of *hemoglobin* genes, and each type expanded through gene duplication, followed by independent evolution. In addition, the β39 threonine was mutated to cysteine in the Southern bluefin tuna genome and other tunas, which may help to transport more oxygens for fast and continuous swimming. In summary, we produced a draft genome assembly and predicted improved hemoglobin-related roles for the fast-swimming activities in Southern bluefin tuna.

## Data Availability

The datasets presented in this study can be found in online repositories. The names of the repository/repositories and accession number(s) can be found below: the data of genome assembly used in this work were deposited at NCBI under the bioproject PRJNA843977 and biosample SAMN28775172. Raw sequencing reads were deposited at China National Genebank (CNGB) under the project CNP0000961 with accession numbers CNX0447365∼0447369.
