# Supplementary material for: Whole genome sequencing of the fast-swimming Southern bluefin tuna (Thunnus maccoyii)
Source: Front Genet. 2022 Nov 3;13:1020017. doi: 10.3389/fgene.2022.1020017 (PMC9670116; doi:10.3389/fgene.2022.1020017)
Supplement: Supplementary file 1 [file DataSheet1.docx]

**Table S1**: Summary of the reference genes

| **Organism** | **Gene** | **Abbreviations** | **Accession number** |
| --- | --- | --- | --- |
|  | *Hemoglobin alpha adult 1* | *hbaa1* | ENSDARP00000101541 |
| *Danio rerio*  (Zebrafish) | *Hemoglobin alpha adult 2* | *hbaa2* | ENSDARP00000092484 |
|  | *Hemoglobin alpha embryonic1.1* | *hbae1.1* | ENSDARP00000107930 |
|  | *Hemoglobin alpha embryonic 3* | *hbae3* | ENSDARP00000112265 |
|  | *Hemoglobin alpha embryonic 5* | *hbae5* | ENSDARP00000066382 |
|  | *Hemoglobin beta adult 1* | *hbba1* | ENSDARP00000092488 |
|  | *Hemoglobin beta adult 2* | *hbba2* | ENSDARP00000053076 |
|  | *Hemoglobin beta embryonic 1.3* | *hbbe1.3* | ENSDARP00000097665 |
|  | *Hemoglobin beta embryonic 2* | *hbbe2* | ENSDARP00000066383 |
|  | *Hemoglobin beta embryonic 3* | *hbbe3* | ENSDARP00000055622 |
| *Homo sapiens*  (Human) | *Hemoglobin subunit gamma-2* | *hba1* | ENSG00000130656 |
|  | *Hemoglobin subunit epsilon* | *hbb* | ENSG00000223609 |
|  | *Hemoglobin subunit gamma-1* | *hbd* | ENSG00000213931 |
|  | *Hemoglobin subunit delta* | *hbe1* | ENSG00000086506 |
|  | *Hemoglobin subunit beta* | *hbg1* | ENSG00000244734 |
|  | *Hemoglobin subunit alpha* | *hbg2* | ENSG00000213934 |
|  | *Hemoglobin subunit zeta* | *hbm* | ENSG00000196565 |
|  | *Hemoglobin subunit theta-1* | *hbq1* | ENSG00000206172 |
|  | *Hemoglobin subunit mu* | *hbz* | ENST00000356815.4 |
| *Lepisosteus oculatus*  (Spotted gar) | *Hemoglobin cathodic subunit beta-like* | *hbb* | ENSLOCG00000007827 |
|  | *Hemoglobin embryonic subunit alpha-like* | *hbae* | ENSLOCG00000007756 |
|  | *Hemoglobin subunit alpha-1-like* | *hba* | ENSLOCG00000007868 |
|  | *Hemoglobin subunit alpha-like* | *hba* | ENSLOCG00000007787 |
|  | *Hemoglobin subunit alpha-like* | *hba2* | ENSLOCG00000007745 |
|  | *hemoglobin subunit epsilon-like* | *hbe1* | ENSLOCG00000007843 |
|  | *hemoglobin subunit epsilon-like* | *hbe1* | ENSLOCG00000007852 |
|  | *Hemoglobin subunit zeta-like* | *hbz* | ENSLOCG00000007770 |
| *Petromyzon marinus*  (Sea lamprey) | *Globin-2* | *globin-2* | ENSPMAG00000007266 |
| *Danio rerio*  (Zebrafish) | *Mahogunin ring finger 1b* | *mgrn1b* | ENSDARP00000072683 |
|  | *Arylalkylamine N-acetyltransferase 2* | *aanat2* | ENSDARP00000002650 |
|  | *Rhomboid family member 1a* | *rhbdf1a* | ENSDARP00000053094 |
|  | *Methylpurine-DNA glycosylase* | *mpg* | ENSDARP00000119099 |
|  | *Nitrogen permease regulator-like 3* | *nprl3* | ENSDARP00000039240 |
|  | *KN motif and ankyrin repeat domain-containing protein 2* | *kank2* | ENSDARP00000128469 |
|  | *Dedicator of cytokinesis 6* | *dock6* | ENSDARP00000077379 |
|  | *Mahogunin ring finger 1a* | *mgrn1a* | ENSDARP00000140478 |
|  | *Forkhead box j1b* | *foxj1b* | ENSDARP00000107064 |
|  | *Rhomboid 5 homolog 1b* | *rhbdf1b* | ENSDARP00000127302 |
|  | *Aquaporin-8* | *aqp8* | ENSDARP00000066381 |
|  | *Leucine carboxyl methyltransferase 1* | *lcmt1* | ENSDARP00000072402 |
|  | *Rho GTPase-activating protein 17a* | *arhgap17a* | ENSDARP00000099577 |

**Table S2**: Accession numbers of the representative fish genomes

| **Scientific Name** | **Common Name** | **Accession number** |
| --- | --- | --- |
| *Gadus morhua* | Atlantic cod | GCA_902167405.1 |
| *Dicentrarchus labrax* | European seabass | GCA_000689215.1 |
| *Seriola dumerili* | Greater amberjack | GCA_002260705.1 |
| *Larimichthys crocea* | Large yellow croaker | GCF_000972845.1 |
| *Mola mola* | Ocean sunfish | GCA_001698575.1 |
| *Thunnus orientalis* | Pacific bluefin tuna | GCA_021601225.1 |
| *Istiophorus platypterus* | Sailfish | GCA_016859345.1 |
| *Lepisosteus oculatus* | Spotted gar | GCF_000242695.1 |
| *Xiphias gladius* | Swordfish | GCA_016859285.1 |
| *Hippocampus comes* | Tiger tail seahorse | GCA_001891065.2 |
| *Cynoglossus semilaevis* | Tongue sole | GCF_000523025.1 |
| *Seriola aureovittata* | Yellowtail amberjack | GCA_021018895.1 |
| *Danio rerio* | Zebrafish | GCF_000002035.6 |

**Table S3**: Summary of the sequenced reads for genome assembly of the Southern bluefin tuna

| **Insert Size** | **Reads Length** | **Raw Data (Gb)** | **Clean Data (Gb)** |
| --- | --- | --- | --- |
| 270 bp | 150 bp | 59.05 | 52.68 |
| 500 bp | 150 bp | 25.56 | 23.74 |
| 800 bp | 125 bp | 28.79 | 26.82 |
| 2 kb | 150 bp | 24.76 | 13.83 |
| 5 kb | 150 bp | 35.03 | 18.73 |
| **Total** |  | **173.19** | **135.8** |

**Table S4:** 17-mer depth distribution and estimated genome size

| **kmer**  **length** | **kmer num** | **Kmer depth** | **genome size** | **used base** | **used read** | **Coverage** |
| --- | --- | --- | --- | --- | --- | --- |
| 17 | 44,309,018,345 | 52 | 852 | 50,496,822,825 | 386,737,780 | 63.8204 |

**Table S5:** Statistics of the genome assembly

|  | **scaffold** | **contig** | **gap** |
| --- | --- | --- | --- |
| Total_length | 806,538,915 | 794,343,312 | 12,171,831 |
| Total_number | 95,979 | 1,564,055 | 32,019 |

**Table S6**: Annotation of repeat sequences in the assembled genome

| **Type** | **Repeat Size (bp)** | **% of genome** |
| --- | --- | --- |
| Trf | 17,799,248 | 2.206868 |
| Repeatmasker | 51,149,429 | 6.341843 |
| Proteinmask | 23,721,168 | 2.941106 |
| *De novo* | 194,507,419 | 24.116309 |
| **Total** | **236,097,722** | **29.272949** |

**Table S7**: Detailed classification of repeat sequences in the assembled genome

|  | **Repbase TEs** | | **TE Protiens** | | ***De novo*** | | **Combined TEs** | |
| --- | --- | --- | --- | --- | --- | --- | --- | --- |
| **Type** | **Length (bp)** | **Percentage** | **Length (bp)** | **Percentage** | **Length (bp)** | **Percentage** | **Length (bp)** | **Percentage** |
| DNA | 28,220,669 | 3.50% | 3,555,207 | 0.44% | 63,952,998 | 7.93% | 79,590,796 | 9.87% |
| LINE | 18,751,030 | 2.32% | 17,163,997 | 2.13% | 23,603,023 | 2.93% | 40,650,413 | 5.04% |
| SINE | 1,251,942 | 0.16% | 0 | 0 | 2,030,127 | 0.25% | 2,256,217 | 0.28% |
| LTR | 7,729,188 | 0.96% | 3,010,574 | 0.37% | 4,480,184 | 0.56% | 13,320,396 | 1.65% |
| Other | 11,631 | 0.14% | 0 | 0 | 0 | 0 | 11,631 | 0.001% |
| Unknown | 0 | 0.00% | 0 | 0 | 101,839,026 | 12.63% | 101,839,026 | 12.63% |

**Table S8:** Summary statistics of gene annotation

| **Method** | | **Software/**  **gene set** | **number** | **average transcript length(bp)** | **average CDS length(bp)** | **average Exons per Gene** | **average exon length(bp)** | **average Intron length(bp)** |
| --- | --- | --- | --- | --- | --- | --- | --- | --- |
| ***De novo*** | Augustus | | 25,717 | 14,997.76 | 1,581.49 | 9.04 | 174.90 | 1,668.26 |
|  | Genscan | | 35,563 | 15,308.06 | 1,490.37 | 8.25 | 180.74 | 1,906.98 |
| **Homolog** | *Danio rerio* | | 20,495 | 11,759.38 | 1,566.08 | 8.50 | 184.15 | 1,358.34 |
|  | *Oryzias latipes* | | 24,727 | 9,299.11 | 1,332.75 | 7.35 | 181.16 | 1,253.25 |
|  | *Oreochromis niloticus* | | 25,760 | 11,242.08 | 1,489.93 | 8.28 | 179.85 | 1,338.76 |
|  | *Takifugu rubripes* | | 21,405 | 11,670.51 | 1,533.89 | 8.57 | 178.79 | 1,337.42 |
|  | *Tetraodon nigroviridis* | | 9,773 | 11,506.38 | 1,519.96 | 8.82 | 172.37 | 1,277.40 |
|  | *Gasterosteus aculeatus* | | 25,956 | 9,670.28 | 1,317.72 | 7.42 | 177.52 | 1,300.45 |
|  | *Gadus morhua* | | 24,056 | 9,516.67 | 1,273.65 | 7.15 | 178.08 | 1,339.88 |
| **Transcriptome** |  | | 22,522 | 8,808.83 | 2,156.77 | 7.44 | 289.79 | 1,032.52 |
| **GLEAN** |  | | 23,403 | 17,660.31 | 1,800.34 | 9.82 | 183.39 | 1,798.79 |

**Table S9:** Statistics of gene functional annotation

| **Database/Parameter** | **Number** | **Percentage (%)** |
| --- | --- | --- |
| Total | 23,403 |  |
| InterPro | 21,290 | 90.971243 |
| GO | 16,458 | 70.324317 |
| KEGG | 19,611 | 83.796949 |
| Swissprot | 20,272 | 86.621373 |
| TrEMBL | 22,253 | 95.0861 |
| Annotated | 22,485 | 96.077426 |
| Unannotated | 918 | 3.922574 |

**Table S10:** All tuna genomes available from NCBI

| **Scientific name** | **Common name** | **Genome Size** | **GC content** | **Complete BUSCOs** | **Repeats content** | **Sequencing technology** | **Accession** | **Reference** |
| --- | --- | --- | --- | --- | --- | --- | --- | --- |
| *T. maccoyii* | Southern bluefin tuna | 806 Mb | 39.0% | 95.8% | 29.27% | Illumina | GCA_025435535.1 | This study |
|  |  | 782 Mb | 39.7% | 98.4% | 26.07% | PacBio;10X; Hi-C | GCA_910596095.1 |  |
| *T. thynnus* | Atlantic bluefin tuna | 944 Mb | 39.5% | 36.9% | N/A | Illumina | PRJNA843977 | Puncher et al., 2018 |
| *T. orientalis* | Pacific bluefin tuna | 827 Mb | 39.7% | 97.0% | N/A | PacBio; 10X | GCA_021601225.1 |  |
|  |  | 787 Mb | 39.6% | 88.2% | 25.25% | NextSeq; PacBio | GCA_009176245.1 | Suda et al., 2019 |
|  |  | 740 Mb | 39.7% | 87.7% | N/A | 454; Illumina | GCA_000418415.1 | Nakamura et al., 2013 |
| *T. albacares* | Yellowfin tuna | 792 Mb | 39.7% | 98.4% | 26.27% | PacBio; 10X; Hi-C | GCA_914725855.1 |  |
|  |  | 728 Mb | 39.5% | 89.2% | 27.8% | Illumina | GCA_900302625.1 | Barth et al., 2017 |

**Table S11:** Summary of the codons at position 39 of β hemoglobin genes in twenty-seven species

|  | MN cluster | | | | | | LA cluster | | |
| --- | --- | --- | --- | --- | --- | --- | --- | --- | --- |
|  | ACT | ACC | ACG | ACA | TGC | TGT | ACT | ACC | TGT |
| *Lepisosteus oculatus* | 1 | 3 |  |  |  |  |  |  |  |
| *Danio rerio* | 4 | 2 |  |  |  |  | 2 |  |  |
| *Gadus morhua* | 1 |  | 1 |  |  |  |  |  |  |
| *Lampris incognitus* | 3 | 1 |  |  |  |  |  |  |  |
| *Gephyroberyx darwinii* |  |  |  |  |  |  |  |  |  |
| *Acanthochaenus luetkenii* |  | 1 |  | 1 |  |  | 3 |  |  |
| *Myripristis murdjan* | 2 | 3 |  |  |  |  | 2 |  |  |
| *Lucifuga dentata* | 1 |  |  |  |  |  | 1 |  |  |
| *Thalassophryne amazonica* |  |  | 2 | 1 |  |  | 1 |  |  |
| *Siphamia tubifer* | 4 |  |  |  |  |  |  |  |  |
| *Boleophthalmus pectinirostris* |  |  |  |  |  |  | 1 |  | 1 |
| *Periophthalmus magnuspinnatus* |  |  |  |  |  |  | 1 |  |  |
| *Periophthalmus modestus* |  |  |  |  |  |  | 1 |  |  |
| *Thunnus maccoyii* |  |  |  |  | 7 | 1 | 1 |  |  |
| *Thunnus maccoyii*  *(from NCBI)* |  |  |  |  | 7 | 1 | 4 |  |  |
| *Thunnus albacares* |  |  |  |  | 8 |  | 8 |  |  |
| *Thunnus orientalis* |  |  |  |  | 5 |  | 2 |  |  |
| *Hippocampus comes* |  |  |  |  |  |  |  | 1 |  |
| *Hippocampus abdominalis* |  |  |  |  |  |  |  | 1 |  |
| *Seriola dumerili* | 5 | 1 |  |  |  |  | 1 |  |  |
| *Seriola aureovittata* | 5 | 1 |  |  |  |  | 1 |  |  |
| *Xiphias gladius* | 6 | 1 |  |  |  |  | 1 |  |  |
| *Istiophorus platypterus* | 5 | 1 |  |  |  |  |  |  |  |
| *Cynoglossus semilaevis* |  |  |  |  |  |  | 2 | 1 |  |
| *Larimichthys crocea* | 4 |  |  |  |  |  | 1 |  |  |
| *Dicentrarchus labrax* | 4 |  |  |  |  |  | 3 |  |  |
| *Mola mola* | 3 |  |  |  |  |  | 1 |  |  |

**Table S12:** A synteny comparison between the two assembly versions of the Southern bluefin tuna genome

| Structural annotations  Variation_type | Count | Length | Length_NCBI |
| --- | --- | --- | --- |
| Syntenic regions | 1,992 | 716,589,361 | 730,445,762 |
| Of our assembled genome |  | 98.1% |  |
| Covering the public assembly |  | 93.92% |  |
| Inversions | 21 | 4,164,315 | 4,380,385 |
| Translocations | 1,310 | 63,747,961 | 64,774,671 |
| Duplications | 408 | 50,847,026 | - |
| Duplications (NCBI) | 2,995 | - | 23,611,847 |
| Not aligned | 2,710 | 24,696,389 | - |
| Not aligned (NCBI) | 3,954 | - | 7,672,231 |
| Sequence annotations |  |  |  |
| Variation_type | Count | Length | Length_NCBI |
| SNPs | 5,388,969 | 5,388,969 | 5,388,969 |
| Insertions | 864,743 | - | 7,252,508 |
| Deletions | 466,721 | 6,198,208 | - |
| Copygains | 225 | - | 971,478 |
| Copylosses | 1,990 | 3,644,742 | - |
| Highly diverged | 27,594 | 20,149,208 | 36,290,206 |
| Tandem repeats | 71 | 108,876 | 144,084 |


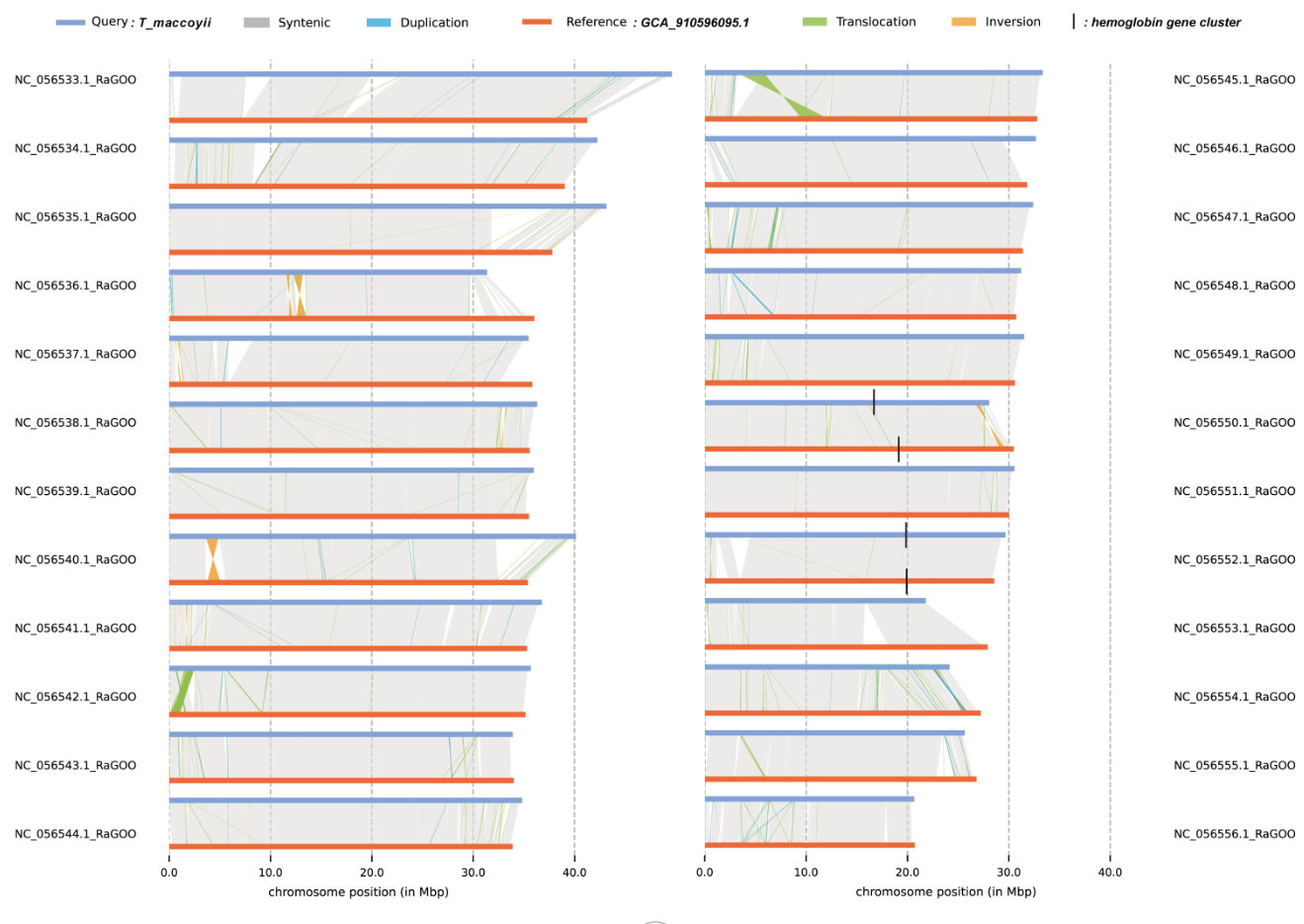


**Figure S1:** A synteny comparison between the two assembly versions of the Southern bluefin tuna genome.


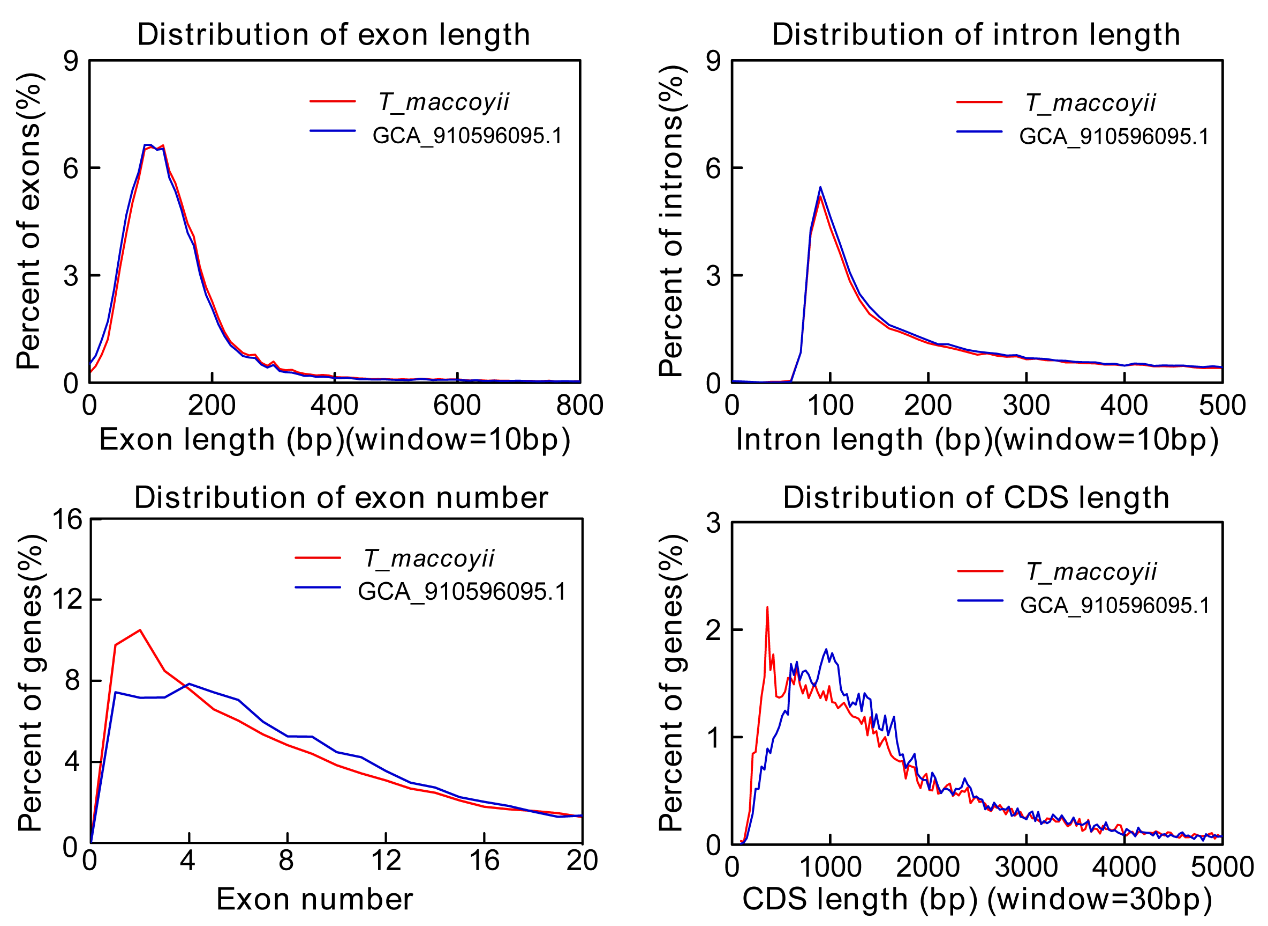


**Figure S2:** Annotation comparison between the two assembly versions of the Southern bluefin tuna genome.


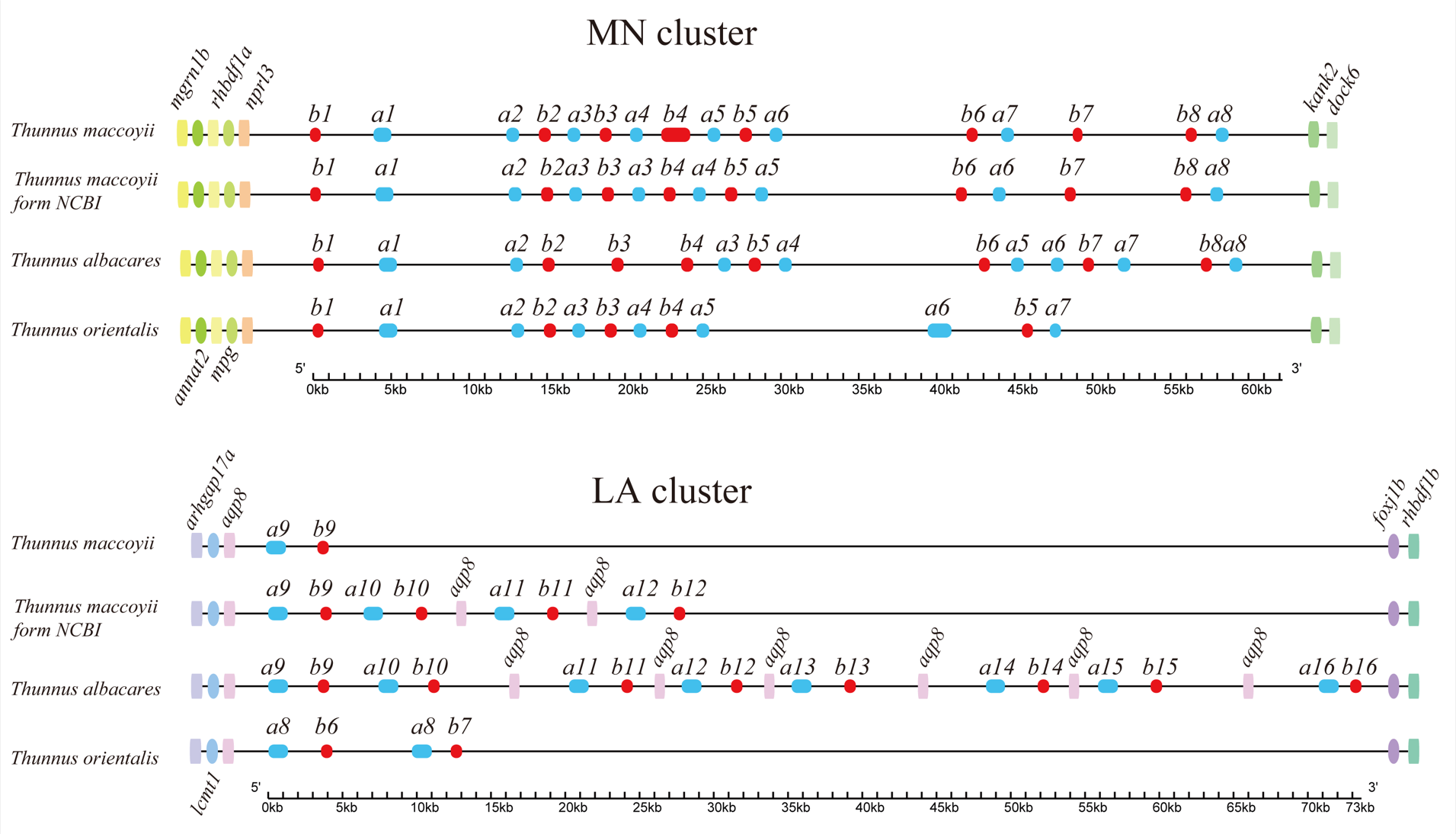


**Figure S3:** Collinearity of hemoglobin genes among genome assemblies of four tunas.


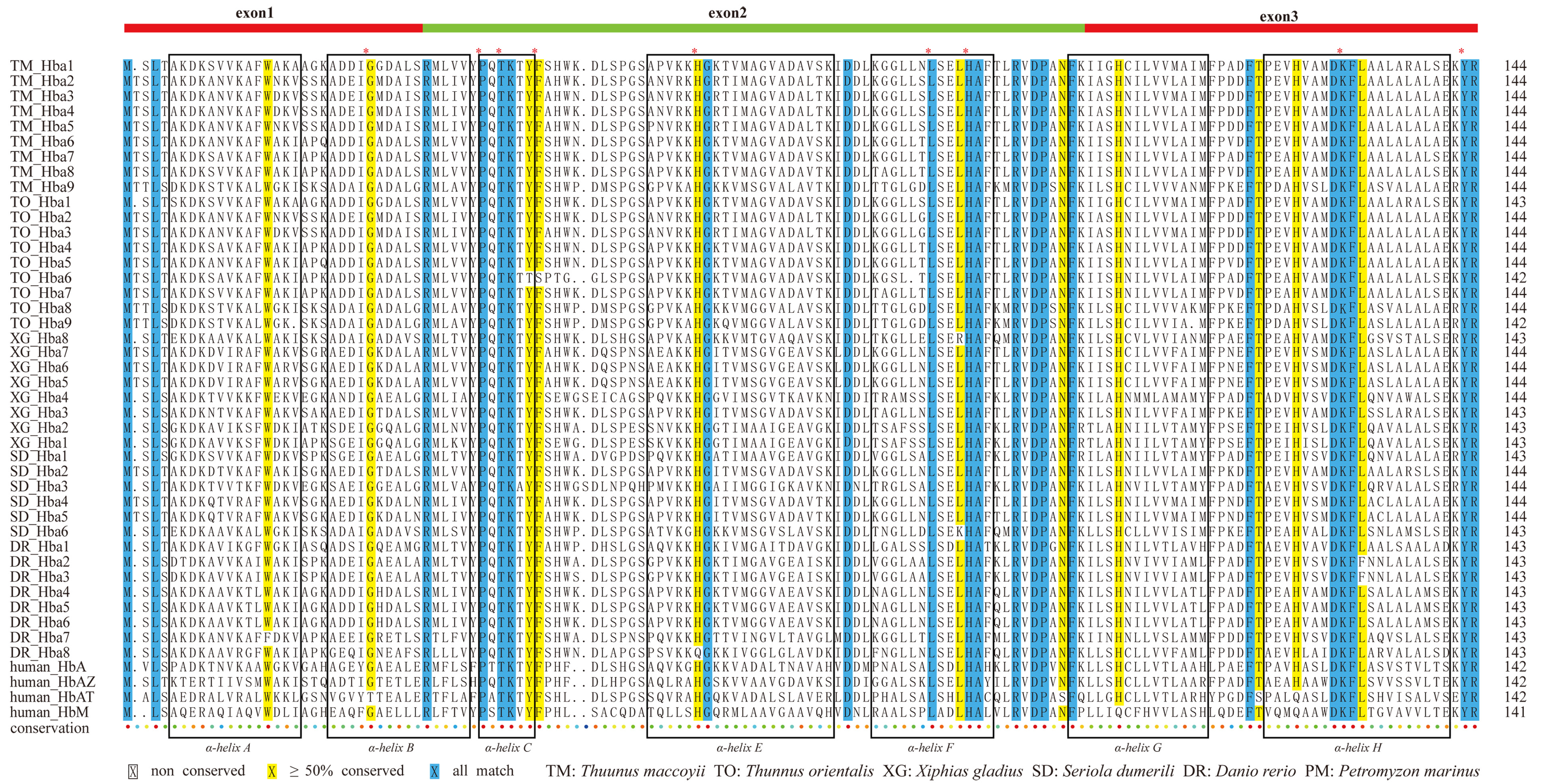


**Figure S4:** ClustalX alignments of α hemoglobins from seven representative species.


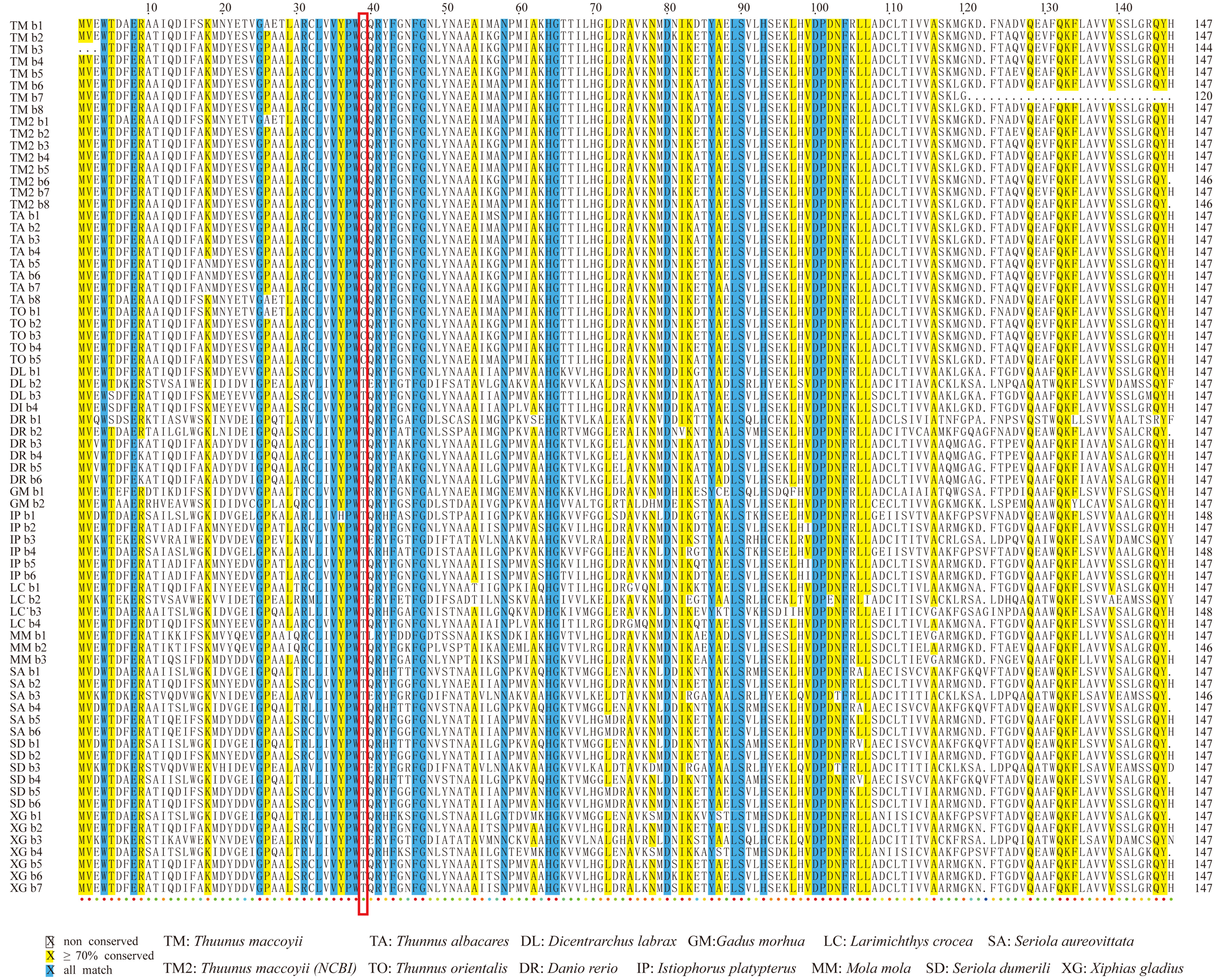


**Figure S5:** Alignments of β hemoglobins in the MN cluster from thirteen fishes.


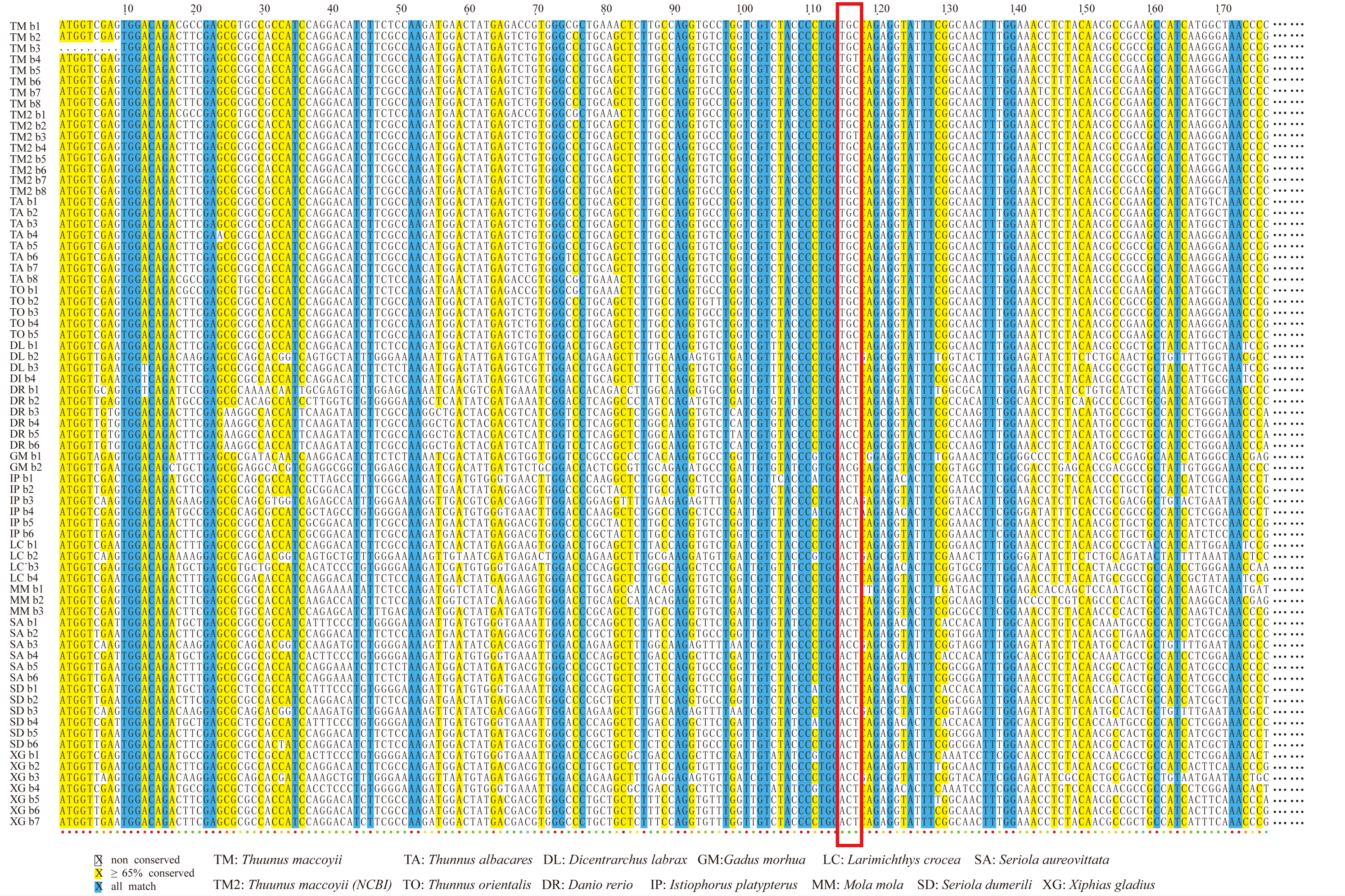


**Figure S6:** Alignments of β hemoglobin coding genes (1-177 nucletide) in the MN cluster from thirteen fishes.
